# THE EFFECTS OF AGE-BASED EVALUATIONS ON OLDER ADULTS’ GAIT VARIABILITY

**DOI:** 10.1093/geroni/igz038.2674

**Published:** 2019-11-08

**Authors:** Sarah Barber, Kate Hamel, Carl Ketcham, Kristy Lui

**Affiliations:** 1 Georgia State University, Atlanta, Georgia, United States; 2 San Francisco State University, San Francisco, California, United States

## Abstract

Older adults are stereotyped as being slow, weak, and frail. In this study we examined how these stereotypes about age-related physical decline affect older adults’ walking performance. Healthy, community-dwelling older adults were asked to walk at their own comfortable pace along a 24’ temporospatial-measuring walkway 10 times. For some participants this was done with a normal-base of support (i.e., usual gait). However, for other participants this was done with a narrow-base of support (i.e., walking within a path of 15 cm outlined by tape). Walking tasks were done either in the presence or absence of a negative age-based evaluation. Results showed that the negative age-based evaluations were associated with greater stride-to-stride variability, particularly for participants who felt less confident in their abilities. Given that gait variability is a predictor of falling, this raises the possibility that negative age-based evaluations can produce concerns that are an intrinsic risk factor for falls.

